# Advance directives in patients with schizophrenia

**DOI:** 10.1017/S1092852924002207

**Published:** 2024-11-27

**Authors:** Jacob M. Appel

**Affiliations:** Psychiatry and Medical Education, Academy for Medicine & the Humanities, Icahn School of Medicine at Mount Sinai, Attending Physician, Mount Sinai Health System, New York, NY, 10027, USA

**Keywords:** advance, directives, schizophrenia, autonomy, psychiary, ethics

## Abstract

Balancing autonomy and beneficence remains an ongoing challenge in the ethical treatment of patients with schizophrenia and other psychiatric disorders of thought. Psychiatric advance directives (PADs) offer one mechanism through which individuals can guide their own care, but unlike medical advance directives, they are not widely utilized in the United States. They are also highly limited by state law in the scope of their legal authority. This article explores the evidentiary basis for PADs as well as the legal and ethical issues that arise in the use of PADs in individuals with schizophrenia, arguing that providers’ fears of complete opt-out from care by patients are likely unfounded and that PADs offer a powerful tool through which individuals with schizophrenia can ensure meaningful consideration of their own values and goals.

## Introduction

The second half of the twentieth century witnessed a “seismic shift towards autonomy in medical ethics” that transformed clinical care and physician-patient relationships.^1^ A widespread consensus emerged in the United States by the late 1970s that patients with decisional capacity should be meaningfully informed about their illnesses and therapeutic options and should possess the authority to manage their own treatment. These principles were notably reflected in the *Belmont Report* (1978), Beauchamp and Childress’s seminal text, *Principles of Biomedical Ethics* (1979), and Dennis Novack’s survey of changing physician attitudes toward divulging patients’ cancer diagnoses (1979).^2^ In contemporary allopathic medicine, empowering capable patients to guide their own treatment has become the nearly universally accepted standard of care. Over the past 50 years, legal mechanisms have also been established to protect the autonomy of patients who have lost decisional capacity. Most notably, advance directive (AD) statutes in all 50 states have enabled capable patients to specify their medical choices for potential times of future incapacitation.^3^ The Patient Self-Determination Act of 1990 compels health care institutions to inform patients about ADs and document their preferences.^1^ The decisions outlined in such documents are generally binding. Patients may specify particular choices through living wills and/or appoint agents, sometimes termed healthcare proxies or health care powers of attorney, to effectuate their preferences during incapacitation. One of the principle goals of such advance directives is to maximize the autonomy of patients by ensuring that, even when they are incapacitated, their care remains consistent with their underlying values and preferences.^4^

For a range of reasons—some artefactual, some logistical, some reflective of a higher concern for safeguarding the rights of individuals with mental illnesses—patients in many jurisdictions have historically not been able to guide their future psychiatric treatment in the same manner as their general medical care. Psychiatric advance (AD) directives, or “psychiatric wills,” proposed in the early 1980s by Thomas Szasz and others, sought to apply the general principles of ADs to patients with psychosis.^5^ Szasz, a leading and controversial critic of nonconsensual psychiatric treatment, even believed that the “use of psychiatric wills might thus put an end to the dispute about involuntary psychiatric interventions.”^6^ In practice, although a significant number of states now have statutes authorizing PADs, and Centers for Medicare & Medicaid Services rules from 2006 require participating hospitals to “comply with these directive,” extraordinarily broad exceptions significantly limit their applicability and utility.^7^ Sociologist Jeffrey W. Swanson and colleagues have noted that current PAD statutes “give doctors wide discretion to ignore them” by allowing patients’ preferences to be overridden without fear of liability in cases where their choices differ from the accepted standard of care.^8^ At the same time, considerable evidence (discussed below) suggests that PADs remain a largely untapped mechanism for addressing the distinctive needs of patients with schizophrenia and other severe disorders of thought. At a minimum, their increased use may prove helpful in distinguishing patients whose psychosis clouds their judgment with regard to treatment choices from those—likely a much smaller number—who possess a sincere and authentic objection to certain forms of life-preserving care. PADs also hold out the promise of empowering individuals with schizophrenia to feel invested in their own medical treatments and to increase their sense of agency with regard to their relationships with physicians and the healthcare system. What follows is a discussion of the legal, evidentiary, and ethical issues that arise in the use of PADs in individuals with schizophrenia.

### Legal background

The terminology on the subject of PAD is evolving and often still lacks consistency. Elizabeth Gallager offers a useful definition of a PAD, also known as a “psychiatric advance statement” in the United Kingdom, as a document that “sets forth a person’s wishes concerning psychiatric treatment in anticipation of the event that he or she may later become incompetent to make informed health care decisions.”^9^ One form of PAD is a Ulysses contract, described by Ryan Spellecy as a document that “enable[s] persons to commit themselves now to a particular course of treatment at a future time if they suspect they will not be willing or able to follow that course of treatment at that future time.”^10^ Similarly, Claire Henderson and colleagues define Ulysses contacts as forms of PADs that “request that care or treatment be given during a future period of incapacity, even over the possible later objection or resistance of the person during a crisis.”^11^ The concept takes its name from an episode in the Homeric epic, *The Odyssey*, in which the title character, Odysseus (Latin: Ulysses) seeks to hear the music of humanlike creatures, Sirens, whose mellifluous songs lure sailors to their deaths. To prevent such a fate, Odysseus orders his sailors to bind him to the mast of his ship, to place beeswax in their own ears, and to ignore his entreaties to release him until the ship has passed the Sirens’ shores—allowing him to enjoy their melodies free from peril. The purpose of Ulysses contracts is to prevent succumbing to what the Ancient Greeks called “Akrasia” or so-called “weakness of the will,” which, in the case of PADs, refers to current preferences that undermine earlier, supposedly more authentic ones.^12^ The following discussion will follow the definitions used by Gallagher, Spellecy, and Harnderson.

American law has historically proven highly unsympathetic to Ulysses contracts. The first known effort to authorize such an agreement in the United States appears to be New York State legislation from 1857 that allowed alcohol use disorder patients to commit themselves voluntarily for 1 year to the New York State Inebriate Asylum. When patient Walter Baker sought to depart before completing this previously approved term, and the facility objected, the New York State Supreme Court ordered him released.^2^ Similarly, in *Ex Parte Lloyd*, a federal district court in Kentucky adopted a constricted view of the Narcotic Farms Act of 1929, also known as the Porter Act, which on the surface seemed to allow substance users to voluntarily enroll themselves in treatment irrevocably. Emery Lloyd, who had agreed to such terms, later asked to be discharged, and the court conceded that he had a constitutional right to be released.^3^ As a general rule, courts until recently viewed Ulysses contracts through the negative lens of contracts for self-enslavement or involuntary servitude and proved unsympathetic.

After Szasz raised the possibility of a “psychiatric will” in 1982, Timothy Howell, Ron Diamond, and Dan Wikler attempted to operationalize PADs with a proposed form.^13^ Starting with Minnesota in 1991 and Hawaii in 1992, states began to enact statutes that authorized PADs.^14^ Yet these statutes were largely designed to allow patients to *agree to* future treatment, not to *reject* future treatment. They arose after a series of court cases from the 1960s through the 1980s that required judicial review before the administration of neuroleptics or ECT to psychiatric patients, delaying care and increasing the administrative burden upon providers, even in situations in which patients did not object to such treatments but only lacked capacity to offer consent. For instance, the “Minnesota advance psychiatric directive statute allows patients to give advance consent to intrusive mental health treatments,” but if the patient refuses such interventions, “Minnesota courts…require…the usual hearings for the forced administration of intrusive mental health treatments.”^15^ That approach has been adopted widely by states that have formally authorized PADs, effectively limiting the scope to Ulysses contracts (i.e., documents that authorize, rather than reject, future care.) Patients can bind themselves to future treatment but cannot reject interventions consistent with the standard of care; rather, in the latter circumstances, they are subject to the underlying laws of their states. For example, New Jersey’s PAD statute is binding except in cases in which honoring the directive would “violate the accepted standard of mental health care or treatment under the circumstances of the patient’s mental health condition,” “violate a court order or provision of statutory law,” or “endanger the life or health of the patient or another person.”^4^ Similarly, Illinois’s PAD statute, the Mental Health Treatment Preference Declaration Act of 1996, contains the caveat that a “declaration does not limit any authority…either to take a person into custody or to admit, retain, or treat a person in a health care facility.”^5^ These statutes, with their broad exceptions, are highly representative of state PAD laws.^16^ Any role they serve in limiting unwanted care likely occurs indirectly, as judges are permitted, albeit not required, to review such documents to guide their decision-making regarding requests for care refusal.

A seminal federal court case, *Hargrave v. Vermont* (2002), called these statutory limits on PADs into question.^6^ That case involved Nancy Hargrave, a woman with a diagnosis of paranoid schizophrenia, who executed a durable power of attorney for health care (“DPOA”) in 1999 in which she stated that she did not wish to receive “any and all anti-psychotic, neuroleptic, psychotropic or psychoactive medications” in the future.^7^ Of note, no available evidence indicated that she lacked capacity to effectuate this document, essentially a PAD, at that time. Her attorneys then sought a court order to prevent any future treatment contrary to this directive. In doing so, they argued that Vermont’s existing PAD statute, Act 114, violated Title II of the Americans with Disabilities Act of 1990. Both the federal district court for Vermont and the Second Circuit Court of Appeals, whose jurisdiction covers the states of Connecticut, New York, and Vermont, agreed—in essence, enabling the enforcement of treatment-rejecting PADs within the state’s boundaries.^8^ ^,^^9^ As Paul Appelbaum explained, “*Hargrave*, then, stands for the proposition that the state, having established a statutory basis for medical advance directives, cannot exclude involuntarily committed psychiatric patients from its coverage.”^17^ Appelbaum warned that the ruling might “chill enthusiasm for psychiatric advance directives among many clinicians” and that *Hargrave*’s legacy may be to inhibit the use of this once-promising tool.”^18^ In contrast, Michael Allen of the Bazelon Center hoped *Hargrave* might prove a “fresh beginning” that would lead to increased “trust building, peer support, talk therapy, and other naturalistic supports” in the patient-psychiatrist relationship and a path for “people with psychiatric disabilities [to] achieve long-term recovery and greater satisfaction with their quality of life.”^19^

In practice, *Hargrave* has not led to meaningful change. That may be, in part, as some claim, because “[a]t a practical level it is most unlikely that many patients who are involuntarily hospitalized…have the knowledge or the resources or the motivation to execute such a will.”^20^ However, one must note that providers and institutions have the ability to provide this knowledge and these resources to patients; data (discussed below) suggests that the motivation already exists but merely remains untapped. At the same time, the ruling has not led to similar cases in other American jurisdictions, nor has the legal landscape evolved significantly on the subject. More than two decades after Nancy Hargrave’s lawsuit, PADs remain underutilized and subject to extensive caveats in most states, restrictions that render both their utility and their appeal to patients extremely limited. In short, the potential of PADs to improve the lives of individuals with schizophrenia remains largely untested.

### Evidence base

Despite their limited implementation to date, the evidence supporting the value of PADs is increasing.^21^ A systematic review and meta-analyses conducted by Emma Molyneux and colleagues reported that PADs can reduce such involuntary commitments by 25%.^22^ Similarly, another meta-analysis by de Jong et al. found “advance statements,” which included PADs and crisis plans, to reduce involuntary commitments by 23%—more effective than community treatment orders. ^23^ The expanded use of PADs outside the United States affords a valuable source of data in this regard.^24^ For instance, a multicenter French study led by Aurélie Tinland found that among patients with schizophrenia, schizoaffective disorder, and bipolar I disorder, PADs facilitated by peer workers led to “significantly fewer compulsory admission.”^25^ Beyond evidence for efficacy, PADs remain highly popular among stakeholders. An analysis by Scholten et al., using “comparisons between the empirical findings…using a structured expert consensus process, found that stakeholders from three European nations expressed meaningful support for the use of PADs and Ulysses contacts in particular.^26^ They reported that “stake-holders did not confirm the fundamental ethical and legal concerns raised by ethicists and legal scholars” and voiced few or no worries about an increase of coercion or the invalidity of SBDs due to a lack of identity between past and present self or outdated consent.”^27^

Joint crisis plans, advance directives negotiated by patients and providers that share many attributes of PADs, have shown promise in enhancing the physician-patient relationship in the United Kingdom, France, and Germany.^28^ ^-^^31^ Benefits of PADs have also been recorded in low resource settings, such as the Indian state of Tamil Nadu.^32^ Moreover, broader research on the value of enhanced autonomy for psychiatric patients in treatment outcomes is robust. As noted by Debra S. Srebnik and John Q. La Fond, “research suggests that having choice and control over important life decisions, such as the selection of treatment or housing, is critical to physical and psychological well-being.”^33^ In contrast, fear of coercion and pressure to relinquish autonomy have been shown to reduce engagement with care among patients with schizophrenia.^34^

The strongest data supporting PADs in the United States derives from the extensive work of Swanson at Duke University. In a large study of 147 PAD completers versus 92 non-completers, with follow-ups at 6 months, 1 year and 2 years, Swanson’s team found that PADs were “significantly associated with fewer coercive crisis interventions” and cut in half the odds of coercive acts such as “transport by police for mental health evaluation, use of handcuffs during transport, involuntary commitment, use of locked seclusion, use of physical restraints in the hospital, and forced medication.”^35^ Michelle Easter, working with Swanson and colleagues, reported the benefits of using PADs with Assertive Community Treatment teams in the United States as well.^36^ Assuming that reductions in coercive interventions are desirable—and no evidence to date suggests a concomitant increase in suicides, violence, or other negative outcomes that might have justified such interventions—this data strongly supports incorporating PADs into routine clinical practice.^37^

Patients with psychiatric illnesses across diverse populations are highly open to the prospect of effectuating PADs.^38^ ^,^^39^ For instance, a study of five groups of stakeholders (including clinicians, administrators, patients, family members and mental health advocates) in the state of Virginia found that more than 90% agreed with the statement that “[a]dvance directives that include mental health care will give people with serious mental health problems more control over their lives” and similar percentages believed that PADs “will lead to a better understanding by providers of what consumers want for treatment in both crisis…and outpatient settings…, and to an improved quality of life for consumers.”^40^ In fact, a meta-analysis conducted by Esther Braun et al. found that while [e]mpirical evidence suggests that PAD completion rates remain very low…[u]sers of mental health services are highly interested in PADs and regard them as tools to improve their involvement in care.”^41^ She also reported that such consumers “generally prefer legally binding PADs that can be revoked only when users are competent to consent.”^42^ Similarly, Marcus Sellers has observed that while “the majority of patients support PADs and would want the opportunity to complete one” and although “family members and clinicians [are] generally…supportive of PADs,” nonetheless “the majority of patients in jurisdictions with the relevant legislation do not complete one.”^43^ This striking disconnect between preference and practice calls not only for a systemic review of the barriers creating this divide but also innovative thinking to explore how PADs might prove most helpful to patients with severe disorders of thought such as schizophrenia.

### A potential path forward

A range of explanations has been offered for the low rates at which PADs are utilized.^44^ These reported barriers include, among others, fear of liability on the part of providers and lack of knowledge on the part of patients.^45^ ^,^^46^ Yet two key related factors that likely play a significant role in the underuse of PADs are providers’ fears of so-called “complete treatment refusals” and patients’ lack of trust that their PADs will be honored.^47^ Clinicians may worry that patients with limited insight will attempt to execute PADs that decline *all* care—although empirical evidence suggests that is not actually the case.^48^ At the same time, a review by Laura Shields and colleagues of 30 studies from a range of countries found that patients are “apprehensive to tell their doctor they have a PAD” and that they suspect “even mentioning the existence of a PAD” might lead to “a negative response from the doctor or involuntary treatment during future hospitalizations.”^49^ The majority of patients believe their preferences will be ignored or overruled.^50^ State statutes that do not permit individuals with mental illnesses to opt out of the standard of care or that constrain the legal authority of PADs are unlikely to reassure psychiatric patients in this regard. In short, patients—whether informed or unwittingly, based upon general suspicions—are accurately responding to the state of the current legal landscape.

This outcome is not inevitable. The ability to accept or reject the standard of care, or even life preserving interventions, need not be an all-or-nothing proposition. Instead, offering individuals with psychiatric illnesses the ability to generate nuanced and even conditional PADs is worth exploration. PADs offer an untapped tool that might benefit patients with schizophrenia in meaningful ways. In the case of Nancy Hargrave, she created such a nuanced PAD, in effect binding herself to long term hospitalization rather than psychiatric medication. Her choice, one must emphasize, might have proven inconvenient for the state and consumed resources, but it did not place her life or the lives of others in direct jeopardy. (Needless to say, funds expended upon Hargrave’s long term institutionalization are funds not spent on the care of others, but whether long-term hospitalization is actually more expensive than the revolving door of treatment-and-release that many patients with schizophrenia endure, even ignoring its existential implications, remains entirely unclear.) Within the context of the resources and options available to her, Hargrave was able to ensure a safe and reasonable outcome more consistent with her own values than the alternative proposed by the state.

Psychiatrists and patients, working together to complete such documents, should they prove binding, might actually increase mutual trust and also drive systemic change. If even a fraction of the 122 000 street homeless individuals in the United States created such a conditional PAD, and these PADs proved binding, one imagines supportive services and scatter-site housing opportunities might expand quickly.^51^

### Ethical considerations

Many ethical challenges that apply to PADs, such as the phenomenon of “bargaining down” and the question of whether the individual who creates an AD is truly the same person subject to one after loss of capacity, apply to all ADs.^52^ For instance, Rebecca Dressler has criticized Ulysses contracts as a form of “self-paternalism.”^53^ A systemic review of 50 articles by Stephenson et al. found concern that Ulysses contracts in particular might “be intended as a tool to increase service user autonomy,” but “would ultimately diminish autonomy” and reported arguments that PADs should be “void and non-enforceable” because patients “would forfeit the very liberty that underlies the validity of the document[s].”^54^ Yet, as noted above, this finding is inconsistent with patients’ own reported concerns.^55^ These are important issues, but they have been addressed extensively elsewhere in the literature and are beyond the scope of this paper.^56^ However, the issue of concerns regarding complete opt-out is unique to behavioral health and requires further consideration.

The debate surrounding PADs too often conceptualized the options as binary: either a patient will accept care or they will refuse *all* treatments. This framing ignores the prospect for considerable space between these two extremes. In fact, evidence shows that many patients view PADs as a mechanism for steering care rather than rejecting it. Although many providers express concerns that fully enforceable PADs will lead large numbers of patients with severe mental illness to forgo all psychiatric care, existing evidence does not support this apprehension.^57^ A meta-analysis by Anne-Sophie Gaillard et al. found that complete care refusals are rare: for instance, across 42 studies, only 0.3% of participants “used their PAD to refuse hospital admission under any circumstance.” Rather, respondents often specified particular interventions they did not want (e.g., “group-based therapy”) or noted limits to when certain interventions were acceptable to them (e.g., ECT only “[w]hen I have suicidal thoughts”).^58^ The fear that large numbers of patients will executive blank refusals is, quite frankly, counterintuitive. Patients executing PADs must meet established decisional capacity standards to do so. These are governed by statute in the vast majority of states.^59^ The number of individuals who meet such capacity standards and wish to make choices that endanger others at the expense of care is likely to be exceedingly small; in addition, since this is volitional behavior in the setting of capacity, such acts—like any other volitional, clear-minded acts of violence—should be matters for the criminal justice system, not behavioral health providers. Similarly, the number of individuals who meet capacity standards and still wish to accept significant risks to their own safety, even when offered effective care, is also likely to be minute. Such unusual cases may involve patients with extensive histories of existential suffering due to severe mental illness who, in periods of full stabilization, make an informed choice not to endure such suffering in the future.^60^ Anyone who works closely with patients with schizophrenia recognizes that such cases are likely to be rare outliers; fears of such complete care refusals by capacitated patients should not stand in the way of using PADs to assist and empower the vast majority of patients with schizophrenia or other severe psychiatric conditions. Moreover, since PADs in the United States operate so that the default is full care—unless a patient has executed a PAD to opt out—the risk of widespread opt out, especially by those most in need of treatment, is minimized even further.

Patients with schizophrenia, when they are stabilized and possess relevant decisional capacity, do not want to reject all care. Rather, what they want is *effective* care. Or care different from, but not inherently worse, than what has previously been offered to them. As important, they want this care in the context of a social safety network that ensures their other basic needs—food, shelter, emotional support—are met. PADs offer patients an opportunity to collaborate with providers to spell out conditions for care that are consistent with basic human dignity and wellbeing. Achieving buy-in may require that states honor PADs to the same extent that they honor other medical advance directives, through which patients with capacity are able to reject unwanted future care—even at the expense of their own safety or criminal sanction. Yet the tradeoff may actually prove to be many lives saved and even more lives improved, as the trust engendered by such collaboration toward PADs will lead increasing numbers of patients with schizophrenia to receive care that is more consistent with their own underlying values and preferences.

## Conclusions

In light of the empirical evidence favoring PADs discussed above, a strong argument exists for following the lead of the Second Circuit Court of Appeals and giving binding effect to PADs—both Ulysses contracts and those that reject various forms of future care. Such legal validity would likely result in many psychiatrists discussing PADs with their patients and even facilitating their completion; beyond the specific benefits of the future guidance offered by these PADs in upholding patients’ autonomy and underlying values, such discussions are likely to improve the therapeutic relationship, patient engagement with care, and overall trust between patients with schizophrenia and the mental healthcare system. Providers may gain a better understanding of their patients’ needs and concerns, while patients themselves may feel heard and empowered.

The embrace of unrestricted PADs is bound to prove controversial. Some critics may even view such an approach as a gamble. As with any other significant reform, changes should be piloted with small populations of patients and studied carefully before being enacted on a larger scale. After all, no policy’s efficacy can ever be guaranteed prior to its implementation. However, based upon extensive existing evidence, the risks from such an intervention appear to be relatively low. In contrast, with 122 000 undomiciled individuals with schizophrenia and other severe psychiatric illnesses living on American streets, the current system is clearly not serving the interests or protecting the welfare of this vulnerable population. Unrestricted PADs offer the prospect for systemic change—and such change is long overdue.

## Footnotes

1 Patient Self-Determination Act, 42 U.S. Code § 1395 cc(f) & 42 U.S. Code § 1396a(w) (1991).

2 In the Matter of Walter Baker, 29 How Prac NY 485 (N.Y. Sup.Ct. 1865).

3 Ex Part Lloyd. 13 E Supp. 1005 (E.D. Ky. 1936).

4 N.J. Stat. § 26:2H-110 (2022).

5 755 ILCS 43 (1996).

6 Hargrave v. Vermont, 340 F.3d 27 (2003).

7 Hargrave v. Vermont, 340 F.3d 27 (2003).

8 Vt. Stat. Ann. tit. 18, § 7624 (2023).

9 42 U.S.C. § 12 101 (2024).
